# From sequencing to intelligence: how AI is transforming metagenomics

**DOI:** 10.7717/peerj.21137

**Published:** 2026-05-04

**Authors:** Fathi A. Mubaraki

**Affiliations:** Faculty of Computers and Information Technology, University of Tabuk, Tabuk, Saudi Arabia

**Keywords:** Metagenomics, Artificial intelligence, Machine learning, Data science, Next-generation sequencing, Shotgun metagenomics, Microbial communities, Deep learning

## Abstract

Microbial communities are critical in advancing human health. Metagenomics is a technique that analyzes these communities and allows for investigating their composition and functions. Metagenomic shotgun sequencing enables to capture all of the genetic material in environmental samples, such as water, soil, or the human gut. Despite this advantage, one of the main challenges of this technique is the assembling and interpreting of its data, as it produces many short, fragmented reads. Though long-read technologies may change this in the future, artificial intelligence (AI), machine learning (ML) and data science (DS) offer a powerful solution now, enabling scientists to efficiently process and analyze these large and complex datasets. This review explores the latest advancements in AI and ML applications across the metagenomic pipeline. First, it examines the impact of deep learning (DL) on next-generation sequencing, particularly for long-read technologies. Then, it discusses how ML is automating and improving quality control processes, as well as the use of AI applications in metagenome-assembled genome (MAG) assembly, with a focus on contig binning. Finally, this article looks at how AI and ML can improve predictive modeling for phenotype prediction.

## Introduction

The Human Microbiome Project studies the various microbial communities that naturally inhabit healthy humans. This project provides a foundational overview of the human microbiome along with its role in health (Huttenhower et al., 2012). It investigates the healthy human microbiome across different body habitats, such as the gut, skin, and vagina. It has found that the composition of microbial communities varies across healthy individuals. Diet and lifestyle can significantly influence the gut microbiome, and in so doing also contribute to various health outcomes.

Numerous studies have been conducted to determine how the gut microbiome changes with host age. Tamayo et al. (2024) investigated how the composition and diversity of the gut microbiome can affect both healthy and unhealthy aging. They found that diet, physical activity, stress and mental health can positively or negatively impact the gut microbiome and the aging process. Badal et al. (2020) highlighted that older adults, especially centenarians, tend to have higher microbial diversity (both alpha and beta) compared to younger adults. The gut microbiota of older adults shows distinct patterns. For example, centenarians (people who live to 100 years of age or beyond) show an enrichment of beneficial microbes, like *Akkermansia*, and a lack of other bacteria, such as *Faecalibacterium* and *Bacteroidaceae*. This diversity is a potential marker of healthy aging and longevity.

Improvements in next-generation sequencing (NGS) technology have enabled the sequencing of microbial communities more comprehensively (both in depth and read length). As a result, metagenomics has become increasingly powerful for understanding these communities. This modern approach enables high-throughput analysis of the collective genome of microbial communities in various environments, including the microbiota in soil, water, the human gut, as well as in understudied environments like the dromedary camel (Mubaraki, 2025). In metagenomics, DNA is extracted from a sample which contains multiple microbes, and then sequenced to identify and analyze the entire microbial community at once (Handelsman, 2004; Hugenholtz & Tyson, 2008; Wooley, Godzik & Friedberg, 2010; Thomas, Gilbert & Meyer, 2012). This method helps researchers understand the diversity, functions, and interactions within microbial communities.

Two common techniques used for the study of microbiomes are 16S ribosomal RNA gene sequencing (16S rRNA) and shotgun metagenomics (community WGS). The 16S rRNA approach requires sequencing a conserved gene found across the majority of bacteria (and archaea) to identify and classify microbes within the sequenced community at the genus, or occasionally species, level (Ranjan et al., 2016; Klindworth et al., 2013; Zhang et al., 2023). 16S rRNA gene sequencing is mainly used for taxonomic profiling and is limited to bacteria and archaea. In contrast, shotgun metagenomics sequences all DNA with a sample. This approach covers bacteria and archaea, but extends to all other organisms in the community as well, including viruses and fungi. This approach offers a comprehensive picture of both microbial taxonomy and function, making it ideal for more detailed studies that require functional analysis and/or strain-level resolution (Quince et al., 2017; Sharpton, 2014; Xie et al., 2023).

**Figure 1:**
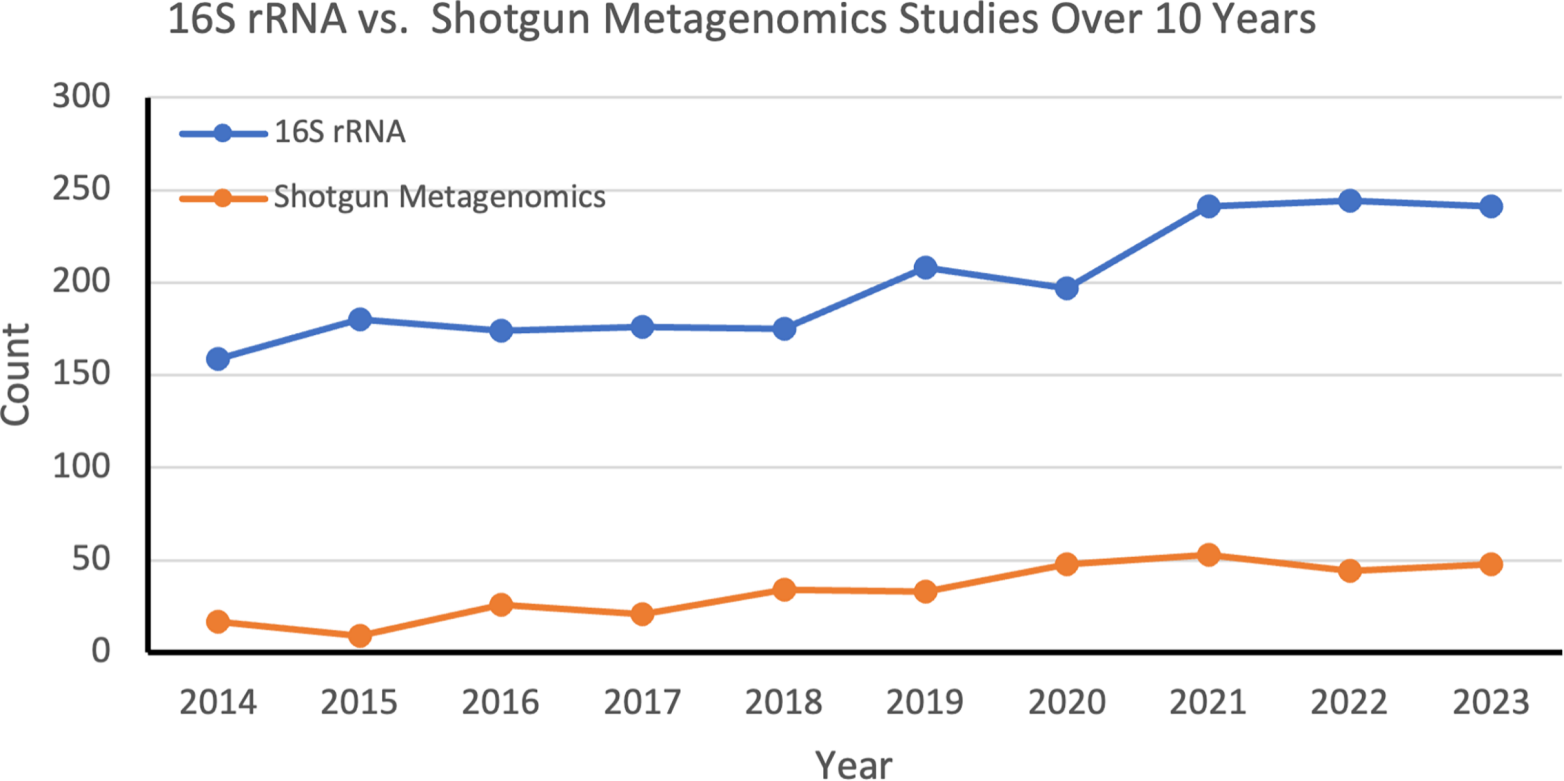
16S rRNA *vs*. shotgun metagenomics studies over 10 years. The number of studies employing the 16S rRNA and shotgun metagenomics methods, as indexed in the PubMed database, from 2014 to 2023.

Figure 1 illustrates the number of studies using 16S rRNA sequencing, compared to the number of studies using shotgun metagenomics, from 2014 to 2023, based on the following search queries:

- (“16S rRNA sequencing”[Title] OR “16S rRNA”[Title]) AND (“2014/01/01”[Date-Publication]: “2023/12/31”[Date-Publication])
- (“shotgun sequencing”[Title] OR “shotgun metagenomics”[Title]) AND (“2014/01/01”[Date-Publication]: “2023/12/31”[Date-Publication])

16S rRNA sequencing was more frequently used throughout this time, and maintained a relatively steady trend, with a slight peak in 2019. Although the use of shotgun metagenomics has grown and continues to do so, it remains less common compared to 16S rRNA sequencing.

In the study of microbial communities, metagenomics is a widely used technique that offers several significant benefits. It enables taxonomic profiling, which identifies and quantifies the abundance of species such as bacteria, fungi, and viruses present in a sample (Shah et al., 2021), essentially answering the question, “Who is there?” While 16S rRNA sequencing provides lower resolution, shotgun metagenomics delivers deeper coverage and reveals the functional potential of microorganisms through functional profiling. It examines genes in the dataset to answer both “Who is there?” and “What can these organisms do?” (Hera et al., 2024).

Artificial intelligence (AI) and machine learning (ML) offer state-of-the-art solutions in metagenomics research. These technologies uncover hidden patterns in complex microbial communities that could be difficult to achieve with traditional univariate statistical approaches. Unsupervised ML, for example, is useful in identifying unknown structures in data without prior knowledge of potential associations. This method has been applied to microbial studies, such as clustering subjects by distinct microbiome subtypes, or partitioning bacterial cell populations based on gene expression profiles, which demonstrates its ability to reveal novel types of information (Asnicar et al., 2024).

The review by Marcos-Zambrano et al. (2021) provides a comprehensive overview of machine learning algorithms applied to microbiome research. It primarily focuses on methodological aspects of ML models and classification tasks. In contrast, this study focuses on the metagenomic pipeline. It covers AI applications across all stages of metagenomic analysis including sequencing technologies, basecalling, quality control, metagenome-assembled genome, and downstream predictive modeling. Furthermore, this review extends to include recent technologies that were not covered in the earlier study such as third-generation sequencing, AI error correction and real-time microbiome analytics. This review highlights how AI is reshaping the entire metagenomics pipeline. Table 1 summarizes the key differences in scope and focus between previous reviews and the present work.

**Table 1:** Comparison between previous reviews and the present work.

Aspect	Marcos-Zambrano et al. (2021)	This review
Primary focus	ML algorithms and their applications in microbiome analysis	AI/ML integration across the entire metagenomic pipeline, from sequencing to prediction
Scope	Feature selection, biomarker identification, disease prediction, and treatment	Sequencing technologies, quality control, assembly, functional profiling, and predictive modeling
Data types	Primarily 16S rRNA and shotgun metagenomics	16S rRNA, shotgun metagenomics, long-read sequencing (PacBio (Eid et al., 2009), ONT (Clarke et al., 2009))
Sequencing technologies	Briefly mentioned: focus on NGS short-read data	Extensive coverage of SRS, LRS, hybrid sequencing, and third-generation technologies
Deep learning for basecalling	Not covered	Covered (*e.g*., DeepConsensus (Baid et al., 2023), MSRCall (Yeh & Lu, 2022), transformer models)
Quality control (QC)	Not a primary focus	Detailed discussion of traditional and AI-based QC for short- and long-read data
MAG assembly	Not a primary focus	Contig assembly, binning (SemiBin2 (Pan, Zhao & Coelho, 2023), VAMB (Nissen et al., 2021)), and quality assessment (CheckM2 (Chklovski et al., 2023))
ML methods coverage	Detailed review of supervised, unsupervised, ensemble methods, deep learning	Overview of ML applications rather than algorithmic comparison

Artificial intelligence has transformed microbiome research. As the field shifts from short-read to long-read sequencing technologies, machine learning has helped improve the accuracy of base-calling in long-read sequencing data, such as Oxford Nanopore and Pacific Biosciences technologies. This advancement ensures higher quality data for downstream analysis. ML also enhances quality control (QC) by automating error detection. Furthermore, AI and ML have advanced metagenome-assembled genome (MAG) assembly by developing more efficient binning and quality assessment tools. They also streamline taxonomic and functional profiling, allowing biologists to quickly identify microbes within a sample, and determine their potential functions. Finally, AI helps build predictive models for disease prediction and proposes personalized treatments based on microbiome data.

**Figure 2:**
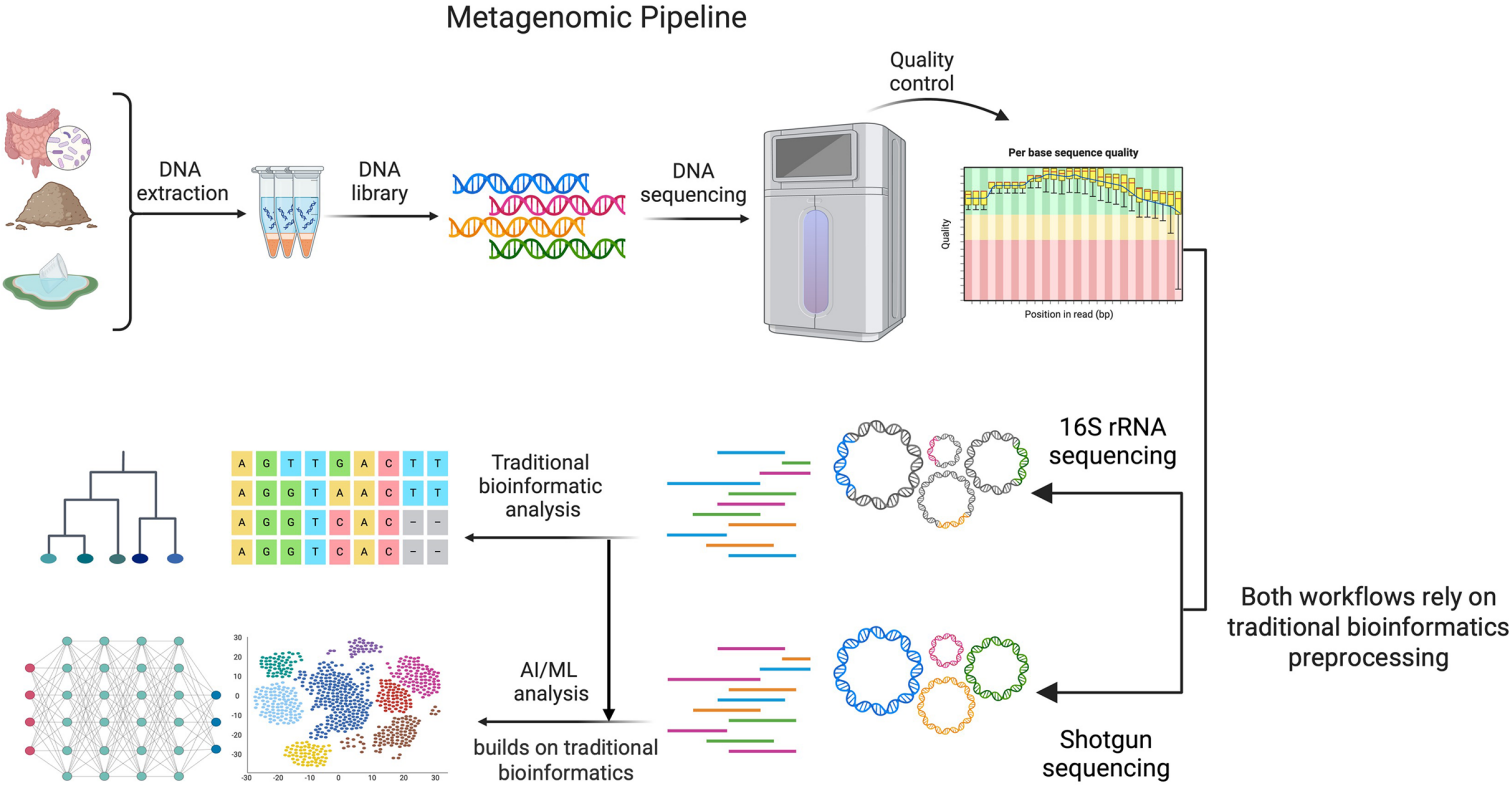
Metagenomic pipeline. The core steps in a metagenomic pipeline are represented here, beginning with initial DNA extraction, then quality control, sequencing (using either traditional 16S rRNA or shotgun metagenomics), and bioinformatics and AI/ML analysis. Although traditional bioinformatic analytical approaches are appropriate for both 16S and shotgun-based metagenomic data, shotgun data provides a much larger feature space (millions of base-pairs per genome *vs* a few hundred, and gene-level annotations). This results in increased potential for deep learning or other advanced AI applications. In both workflows, traditional bioinformatic preprocessing is required, and AI/ML analysis is applied downstream and built on the outputs of these traditional bioinformatic steps. (Created in: BioRender).

Thus, this review explores how these technologies transform each stage of the metagenomics pipeline. Figure 2 illustrates the key steps in this pipeline, and highlights the integration of AI and ML at each stage. In this review, we provide a comprehensive overview of metagenomic analysis that connects traditional metagenomic methods with the emerging applications of AI.

This review is broadly intended for multiple audiences. It is meant to serve as an introduction to modern concepts in metagenomics for new students in the field, in addition to a primer for computational and data scientists to highlight the ways in which microbiologists and microbial ecologists are embracing techniques in machine learning. It also serves as an application note for AI scientists interested in learning how the technologies developed in their field are being applied to a very different one, and as a methods update for established microbiome scientists to showcase new and emerging computational developments in their field which may spur further experimentation and adoption of new AI-enabled methodologies.

## Search methodology

This review explores the intersection of artificial intelligence (AI), data science, and metagenomics. A literature search was conducted using PubMed and Google Scholar in order to identify the studies included in this review. Keywords including machine learning, artificial intelligence, long read, short read, shotgun metagenomics, next-generation, third-generation sequencing, and microbiome were used (530 initial combined search results). Peer-reviewed articles that were published from 2004 to 2024 were focused on, with particular emphasis on recent publications. Non-English articles were excluded from the search. To ensure comprehensive coverage of the field, earlier but highly cited studies were also included when relevant. In total, 73 references were selected for inclusion in this review.

## Metagenomic sequencing methods

Metagenomic sequencing can be divided into short-read, long-read, and hybrid sequencing. Short-read sequencing (SRS), sometimes referred to as next generation sequencing (NGS), has accelerated genetic research. It enables high-throughput analysis at a lower cost compared to traditional Sanger sequencing. Characterized by its ability to massively parallel sequencing of short DNA fragments, typically ranging from 50 to 300 base pairs (Hu et al., 2021; Kumar, Cowley & Davis, 2024; McCombie, McPherson & Mardis, 2019), SRS can generate millions to billions of reads per run. This technology has greatly increased sequencing output. It enabled researchers to tackle complex biological questions involving whole genomes and metagenomes (Jeon et al., 2021). Platforms such as Illumina, MGI, and Ion Torrent are the most commonly used for SRS. Each platform provides unique advantages in terms of throughput, read length, accuracy, and cost-effectiveness (Kumar, Cowley & Davis, 2024; van Dijk et al., 2014; Quail et al., 2012). The high throughput, shorter read lengths, and cost advantages of SRS, as highlighted in Table 2, make it accessible for many genomic applications.

**Table 2:** Comparison of SRS and LRS technologies.

	Short-read sequencing	Long-read sequencing
Throughput	High throughput	Lower throughput
Read length	(~50–300 bp)	10 kb+
Cost	Lower cost	Higher cost
Platforms	Illumina, MGI, Ion Torrent	PacBio SMRT, ONT

Although SRS has significantly advanced the microbiome field, it faces limitations due to its relatively short read lengths. This raises difficulties in resolving repetitive regions and assembling complex genomes accurately (Kumar, Cowley & Davis, 2024; van Dijk et al., 2014). In the context of repetitive regions, the lack of read-level specificity makes it difficult to assign many short reads to a single organism. This problem becomes more obvious when similar species are present in the sample. As a result, more sophisticated bioinformatics algorithms are necessitated by SRS to make sense of the large but comparatively disjointed data generated.

Long-read sequencing (LRS) is sometimes called “third-generation sequencing” (TGS). It allows for the analysis of DNA and RNA molecules in greater detail than short-read methods. Unlike SRS, which relies on the fragmentation of DNA, LRS technologies directly sequence native DNA or RNA molecules. They generate reads that can span tens of thousands of bases and even exceed a million bases in some cases (Amarasinghe et al., 2020; De Coster, Weissensteiner & Sedlazeck, 2021; Logsdon, Vollger & Eichler, 2020). Examples of this method include Pacific Biosciences’ (PacBio) single-molecule real-time (SMRT) sequencing (Eid et al., 2009) and Oxford Nanopore Technologies’ (ONT) nanopore sequencing (Clarke et al., 2009).

As illustrated in Table 2, LRS techniques provide longer read lengths. This ability to sequence long fragments provides distinct advantages over SRS approaches. These include improved *de novo* assembly, more accurate mapping, enhanced identification of transcript isoforms, and increased detection of complex structural variants (Amarasinghe et al., 2020; Satam et al., 2023), which utilize shorter read lengths. Notably, the longer reads produced by LRS are particularly useful for accurately assigning each read to a specific reference organism. They are also valuable for resolving repetitive genomic regions and complex structural variations. In addition, long-read sequencing enables strain-level resolution. It allows researchers to distinguish closely related microbial strains that short reads could not (Afolayan et al., 2024). All of these create challenges for short-read taxonomy assignment and assembly methods.

Despite these advantages, LRS technologies have a lower throughput compared to SRS, as well as higher error rates (Amarasinghe et al., 2020; Warburton & Sebra, 2023; Conlin et al., 2022). LRS platforms often produce fewer reads per unit of time than SRS platforms. Therefore, despite the longer reads afforded by LRS, the total amount of data generated in a single sequencing run may be less than a comparably priced SRS run. Long-read sequencing platforms can generate reads with lengths spanning from tens to hundreds of kilobases, but the longer the read, the more errors it is likely to contain. This is especially true if there are repetitive or homopolymer regions (Tørresen et al., 2019; Zhang, Jain & Aluru, 2020). Another disadvantage is cost. Though the cost of long-read sequencing has significantly decreased over the years, it remains higher than SRS. For example, the cost of PacBio Sequel II HiFi and ONT PromethION remains relatively high. For instance, at comparable sequencing quality to SRS, PacBio Sequel II HiFi sequencing offers highly accurate reads with over 99% accuracy, and generates 25-fold coverage of a human genome. However, this process requires three SMRT Cell 8M which increases the cost by two to three times that required for continuous long-read (CLR) data, which has lower single-pass read accuracies, typically in the 85–90% accuracy range (Logsdon, Vollger & Eichler, 2020).

Recent advances in Oxford Nanopore Technologies, specifically their Kit 14 chemistry, have greatly improved sequencing performance. In tandem with AI-enabled base-calling, these developments are beginning to enable simplex (single molecule, without consensus) accuracy of over 99% per read. This represents an exciting leap in LRS usability. Newer AI models, including a new basecaller using a transformer architecture, are pushing these accuracies even higher with existing chemistries (Oxford Nanopore Technologies, 2024).

**Figure 3:**
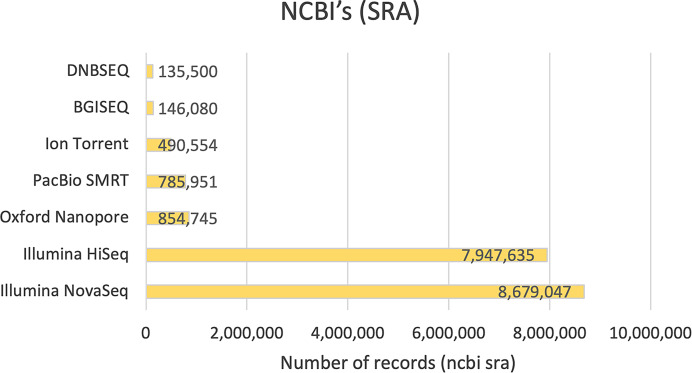
Number of records (NCBI SRA). The usage of various sequencing platforms based on the number of records in NCBI’s SRA, with SRS platforms (HiSeq, NovaSeq, Ion Torrent, BGISEQ, and DNBSEQ) accounting for approximately 92.04% of the total records.

Figure 3 illustrates sequencing platform usage in NCBI’s Sequence Read Archive (SRA) (Leinonen et al., 2011), retrieved on December 21, 2024. It shows Illumina’s dominance, with NovaSeq and HiSeq contributing the most records due to their high throughput and cost efficiency. In contrast, LRS platforms like Oxford Nanopore and PacBio SMRT have fewer records. However, they are essential for applications requiring longer reads, such as *de novo* assembly and structural variation analysis. Their lower usage reflects higher costs and lower throughput.

Hybrid sequencing combines data from both long-read and short-read DNA sequencing technologies to leverage the strengths of each. This approach aims to produce more accurate and complete genomic assemblies (De Maio et al., 2019). Hybrid assembly may be more advantageous than long-read-only assemblies for complex applications like metagenomics and single-cell genomics. One such tool that demonstrates the power of hybrid assembly is Unicycler, a hybrid assembly tool designed specifically for bacterial isolated genomes. It combines the accuracy of SRS with the structural resolve of LRS to generate high-quality assemblies (Wick et al., 2017). Unicycler begins by constructing an accurate and connected assembly graph from short reads using SPAdes. Then it refines this graph by aligning long reads to identify the best paths. This approach is also reflected in algorithms like HybridSPAdes, which combine the precision of short-read from platforms like Illumina with the coverage provided by long-read such as SMRT and nanopore sequencing. The hybrid method efficiently assembles genomes even with low coverage of long reads, thereby significantly reducing sequencing costs (Antipov et al., 2016).

## Deep learning for base-calling

Oxford Nanopore and Pacific Biosciences (ONT and PacBio) have both recently integrated deep learning (DL) techniques to improve the accuracy of base-calling during DNA sequencing. These technologies use deep learning in order to better differentiate between nucleotide possibilities, even when the raw signals might be weak or ambiguous. A recent study by Baid et al. (2023) presented a new method called DeepConsensus, which uses advanced deep learning models to improve the accuracy of DNA sequencing with Pacific Biosciences’ technology. DeepConsensus improves on older methods like pbccs by reducing sequencing errors. The sequences it reads are more accurate, making it easier to assemble a complete and correct genome (Baid et al., 2023). For instance, when compared to the traditional pbccs, DeepConsensus significantly reduced read errors by 42%. This improvement increased the quality of the HiFi reads across different quality levels: Q20 by 9%, Q30 by 27%, and Q40 by 90%.

DeepConsensus uses a transformer–encoder, a type of deep learning (DL) model, to correct mistakes it reads in the DNA sequences. This method is shown to increase the quality and quantity of accurate reads, making the sequencing data more reliable. As a result, it improves the genome assembly—boosting the NG50 contiguity from 4.9 megabases (Mb) to 17.2 Mb and increasing gene completeness from 94% to 97%. It also reduces the false gene duplication rate from 1.1% to 0.5%. In addition, it improves assembly base accuracy from Q43 to Q45, and decreases variant-calling errors by 24% (Baid et al., 2023). Furthermore, because DeepConsensus can accurately handle longer DNA sequences, it enables scientists to study more complex genetic areas, such as metagenomics and biodiversity within bacterial communities. The study suggested that the methods developed for DeepConsensus could be adapted for other research tasks, potentially revolutionizing the study of genetic variation and interactions within and between species.

Another article introduced MSRCall, a new deep learning model designed to improve how the MinION device reads DNA sequences (Yeh & Lu, 2022). The MinION, made by Oxford Nanopore Technologies, is notable for its portability and ability to deliver long DNA readouts quickly (Jain et al., 2016). However, it faces challenges with accuracy, because the signals it reads are complex and noisy. The MSRCall model uses DL techniques, including a multi-scale structure with convolutional and recurrent layers. It combines different layers that analyze the signals at multiple scales, which helps in recognizing both immediate and distant patterns in the DNA strands. This approach allows MSRCall to be more accurate than previous methods. For example, during testing, MSRCall demonstrated better performance compared to the traditional SACall method (Huang et al., 2022). It increased read accuracy from approximately 91% to 92.215% and boosted consensus accuracy from around 99.719% to 99.952%.

At a recent community meeting in Houston, Texas, Oxford Nanopore Technologies announced major improvements in DNA and RNA sequencing. They achieved a new high in sequencing accuracy, with results showing 99.8% accuracy for individual DNA molecules (single sequencing reads). The longest read reached 99.9% accuracy over 1.1 Mb (Oxford Nanopore Technologies, 2023) using a deep learning AI model based on the transformer architecture.

Building on these advancements, the integration of AI and DL has transformed sequencing technologies, especially in metagenomic studies. Microbial ecology and microbiome study greatly benefit from the increased accuracy and information content that AI methods like DeepConsensus can provide. These advances can in turn help in identifying novel microbes, tracking microbial evolution, studying microbial interactions in their natural environments, and more, including the recovery of low-abundance genomes (in assembly) and strain-level resolution (in profiling), thereby improving understanding of the microbiota.

## Metagenomic pipeline

### Quality control

#### Traditional QC (short reads)

Quality control (QC) is an important early step in the sequencing process, as it ensures that the data at the beginning of the analysis funnel is sufficiently accurate or reliable before using it for further analysis downstream. Although many tools have been developed to implement or improve upon QC for sequencing, most of these tools are intended for use with short-read sequencing (SRS) and are not well-suited for third-generation (long-read, LRS) sequencing. There is hence a need for new or improved QC tools.

To illustrate this limitation, several existing tools designed for NGS can be considered. The NGS QC Toolkit (Patel & Jain, 2012) offers comprehensive quality control for sequencing data, while FaQCs (Lo & Chain, 2014) provides rapid evaluation and QC. Other tools, like MultiQC (Ewels et al., 2016), which aggregates and summarizes analysis results from multiple tools and samples into a single report, can help streamline the QC process. Some studies have also demonstrated that effective QC can be achieved even in the absence of a reference genome (Trivedi et al., 2014). Despite their usefulness in certain situations, these tools must be adapted to support third-generation sequencing to remain applicable.

#### Traditional QC (long reads)

A study by Fukasawa et al. (2020) introduced LongQC as an innovative QC tool specifically optimized for third-generation sequencing such as Oxford Nanopore and SMRT sequencing from Pacific Bioscience. This tool excels in automated and efficient QC, covering all major TGS platforms without the need for reference genomes. Similarly, the enhancements to SAMStat 2, as detailed by Lassmann (2023), provided significant improvements, including support for paired-end and long-read data. This expands its use to newer sequencing technologies, allowing the tool to handle more data types and improving its usefulness in sequencing QC. However, LongQC relies on k-mer based approaches. While these are computationally efficient, they might miss the errors that DL models could identify by learning from complex patterns in the data. Similarly, SAMStat 2 still primarily focuses on extracting and visualizing quality metrics rather than predictive models.

#### AI for short-reads QC

Al-Ghalith et al. (2018) published SHI7, a QC pipeline initially optimized for SRS. Notably, this pipeline automatically adjusts parameters used by the QC process based on quality parameters inferred from the sequencing data itself, enabling support for datasets from different sequencing technologies without requiring extensive user input. SHI7’s learning mode autonomously determines the best QC parameters for each dataset. It makes decisions on adapter trimming, quality and length filtering, and whether it is feasible to stitch reads together. Recently, SHI7 was expanded to be compatible with long-read sequences, broadening its utility across sequencing platforms (SHI7 GitHub repository; available at https://github.com/knights-lab/shi7, accessed 7 April 2026).

Albrecht et al. (2021) developed SeqQscorer, a software tool that applies ML to enhance QC processes for NGS data. Using tree-based and DL algorithms, SeqQscorer automates QC by evaluating NGS data quality, thereby improving efficiency. In their study, the authors analyzed 2,642 NGS files from the ENCODE data portal and systematically benchmarked ML algorithms to construct precise, unbiased predictive models. These models, validated across diverse species, demonstrate SeqQscorer’s accuracy. Additionally, Jiajin Li and colleagues introduced ForestQC (Li et al., 2019), a tool that employs Random Forest models (Breiman, 2001) to improve genetic data QC. It evaluates sequencing quality indicators, like depth and genotyping quality, to distinguish between high- and low-quality variants.

#### AI for long-read QC

Herro is a deep learning model designed to correct errors in Oxford Nanopore Technologies (ONT) simplex ultra-long sequencing reads (Stanojević et al., 2024). It enables telomere-to-telomere (T2T)-phased genome assemblies without the need for multiple sequencing technologies. Herro can improve accuracy by up to 100-fold. It supports both R9.41 and R10.4.1 ONT Simplex reads and integrates with *de novo* assemblers like Hifiasm and Verkko. To ensure a high-quality final genome, it utilizes consensus polishing, which serves as its final step. In this process, a draft assembly is optimized using all reads to correct the reference assembly rather than individual reads. This step builds greater confidence in the final result. Tools like Medaka (Oxford Nanopore Technologies, 2025b), which now includes a neural network consensus caller for bacteria and metagenomes, and Dorado Polish (Oxford Nanopore Technologies, 2025a), recently released by ONT, use advanced neural networks to achieve highly accurate polished assemblies.

### MAG assembly: contig, binning, and quality assessment with AI

Reconstructing individual microbial genomes from metagenomic data is a crucial step, especially when the reference databases are limited, or when strain-level and/or genome-resolved functional profiling is desirable. Contigs are continuous DNA sequences assembled from shorter overlapping reads. These contigs are grouped into bins through a process called binning (Du & Sun, 2022). This can be challenging due to the presence of repetitive, and highly conserved regions as well as related organisms within the same sample. Traditionally, for both short and long-read datasets, tools like metabat 2 (Kang et al., 2019) were used. These tools use clustering approaches to group contigs (partially assembled DNA that are not complete genomes) by their tetranucleotide frequency and abundance. AI and ML are powerful tools that may greatly help with this task.

Applying machine learning techniques helps improve the reconstruction of individual genomes from complex metagenomic data. In a recent study, Pan, Zhao & Coelho (2023) introduced SemiBin2, a self-supervised learning tool for both short- and long-read datasets. SemiBin2 achieves high binning accuracy while using less time and memory than SemiBin1. It also performs better than VAMB, a deep learning–based metagenomic binning tool (Nissen et al., 2021). For long-read datasets, it can reconstruct nearly complete bins. These advances make SemiBin2 one of the most reliable tools for metagenomic analysis.

Despite these advances, even state-of-the-art binning algorithms have their limits. Evaluating the quality of metagenomic assembled genomes (MAGs) after the contig assembly and binning processes is essential. This quality assessment estimates the completeness and contamination of each bin or MAG (as a percentage). It allows downstream QC to ensure that the genomes are complete and free from contamination to preestablished thresholds (such as the MiMAG criteria) (Bowers et al., 2017). This process helps confirm that the assembled genome data are more reliable for further analysis.

CheckM2 (Chklovski et al., 2023) is a machine learning tool built to assess the quality of microbial genomes, especially MAGs. It can predict bacterial and archaeal genome completeness and contamination without relying on taxonomic information. CheckM2 uses artificial neural networks (NNs) and gradient boosted (GB) decision trees, which can quickly and accurately evaluate MAG quality. These models are trained on simulated genomes (synthetic MAGs) generated from known prokaryotic reference genomes to predict completeness and contamination, based on KEGG-annotated protein features (Kanehisa & Goto, 2000). It also reports features like genome length and number of coding sequences. The authors showed that CheckM2 performs well in predicting completeness and contamination of genome quality.

## AI and machine learning in microbiome studies: exploration and prediction

### AI/ML for visualizing microbiome data

The field of metagenomic research deals with complex, high-dimensional datasets. These datasets can contain thousands of different bacterial species along with their gene annotation patterns. Traditional methods like principal component analysis (PCA) (Pearson, 2010) can struggle with high-dimensional compositional data because, as a linear method, they fail to effectively capture non-linear relationships.

In contrast, DNABERT-S (Zhou et al., 2025) is a deep learning model developed to classify and group DNA sequences by species, even when reference genomes are not available. It converts DNA sequences into numerical representations, allowing the grouping of sequences from the same species in a shared space. This process helps the model identify unique sequence patterns that distinguish DNA from different species. The model uses two key techniques. Manifold Instance Mixup combines hidden features to generate more challenging training examples, while Curriculum Contrastive Learning trains the model iteratively from simple tasks to progressively more complicated ones. DNABERT-S, per the authors, performs well at species clustering, classification, metagenomics binning, and other DNA discrimination tasks, which makes it a useful tool for analyzing complex DNA data.

DNABERT-S models also serve as a proof of concept as to how AI can address long-standing problems in metagenomics, from species-specific DNA foundational models to binning and classification. AI methods provide a means to advance the state of the art beyond traditional methods and make it easier to study microbial communities more accurately and efficiently.

### AI/ML for phenotype prediction

Predictive modeling in microbiome research involves using computational techniques such as ML to model, estimate, or predict outcomes of biological systems. It is arguably the most critical component of an analysis because it helps identify relationships between microbes and diseases. It also feeds the field’s understanding of microbial roles in the environment. Hernández Medina et al. (2022) highlighted how ML techniques, like Random Forests (RFs) and regression models, are applied to microbial classification, and phenotype prediction. They also showed how recurrent neural networks (RNNs) have been applied to predict changes in microbial communities over time and their association with specific phenotypes. To illustrate this, they referenced the work of Metwally et al. (2019), who created a predictive model based on RNNs that analyzed longitudinal microbiome profiles to predict food allergies in infants over a three-year period.

Beyond association research, a language model has also been developed for the human gut microbiome using natural language processing techniques to capture microbial interactions and patterns. This self-supervised model improves prediction accuracy for conditions like Irritable Bowel Disease (IBD). It achieves this by using deeply embedded hidden features that emerge from the foundation model’s deep understanding of the structure of the human microbiome. This model works well with different datasets and shows how latent features of microbial communities can affect health (Pope et al., 2023). In the opposite vein, a study by Gou et al. (2020) investigated the relationship between gut microbiome features and type 2 diabetes risk using an interpretable ML framework across multiple Chinese cohorts. Its emphasis on interpretable and simplified features distinguishes it from much of the deep learning “black box” models. Instead of relying on hidden representations, it explicitly identifies sets of simpler features with predictive power in a “score.” The microbiome risk score (MRS) correlates strongly with diabetes risk and glucose levels. This study found that body fat distribution and other demographic factors influence the microbiome-diabetes relationship.

Walsh, Stallard-Olivera & Fierer (2023) presented a practical guide to using ML in microbial ecology that emphasizes the importance of exploring and preprocessing microbiome data before modeling. These considerations align with the findings of Marcos-Zambrano et al. (2021), whose study provided a comprehensive review of the most frequently used ML methods in human microbiome research. These include supervised (logistic regression, k-nearest neighbors, naive Bayes, support vector machines) and unsupervised methods such as (hierarchical clustering, biclustering, non-negative matrix factorization). For instance, Random Forests (RFs) are used extensively for classification and biomarker identification due to their ability to handle high dimensionality data. The review mentioned their use in various disease predictions and highlighted their strong performance.

While machine learning models often perform well in prediction tasks, interpretable methods like Shapley Additive Explanations (SHAP) (Lundberg & Lee, 2017) or feature importance rankings from RFs are crucial for gaining biological insights and ensuring the results are meaningful (Walsh, Stallard-Olivera & Fierer, 2023). Marcos-Zambrano et al. (2021) highlighted examples of deep learning applications in microbiome research. One example is the work of Asgari et al. (2018), who used DL methods along with Random Forest and Support Vector Machine (SVM) (Cortes & Vapnik, 1995) to classify human body sites and diagnose Crohn’s disease with high performance in large datasets.

Similarly, they described a study by Fioravanti et al. (2018) that introduced a phylogenetic Convolutional Neural Network (CNN) (Lecun et al., 1998) for classifying gut microbiome data. This model categorized data into six phenotypes. The dataset consisted of 38 controls and 222 IBD patients. Data were pre-processed using QIIME 2 (Bolyen et al., 2019), UCLUST (Edgar, 2010), and RaxML (Stamatakis, 2014) to calculate relative abundance, cluster taxa, and construct a phylogenetic tree as input for the CNN. The CNN outperformed traditional models such as Linear Support Vector Machine (LSVM) and Random Forest in all tasks. This further demonstrates creative uses of ML and AI tools to improve performance in downstream microbiome classification tasks.

### AI/ML for predicting microbial functions

Several studies have applied AI methods to multi-omics data for functional profiling. Wu et al. (2024) showed how metatranscriptomics, metagenomics, and glycomics can be combined with AI to predict diseases. An individual’s gut microbiome can be used as biomarkers for personalized treatment predictions. In this case, machine learning can be applied to predict patterns in a patient and identify microbial biomarkers, which may help improve individual treatments.

A recent study by Odrzywolek et al. (2022) introduced a deep learning model designed to study microbial proteins, particularly when traditional methods fall short due to the novelty and complexity of the data. The model was built using Bidirectional Long Short-Term Memory (BiLSTM) networks (Thireou & Reczko, 2007) and trained on a large database to predict the functions of microbial proteins, especially those absent from existing databases. To validate the model, the researchers tested it on the SwissProt database, a well-established resource for protein information (Bairoch & Apweiler, 2000). Their results showed that the model effectively identifies features related to protein structure and function.

Deep learning is also being used to predict protein structures. One major example is AlphaFold2, developed by DeepMind, which can predict a protein’s 3D shape from its amino acid sequence. New versions like AlphaFold-Multimer and ESMFold can analyze protein complexes and unknown microbial proteins (Levy Karin & Steinegger, 2025). These tools have been applied in several advanced use cases, such as detecting similarity despite low pairwise sequence identity, bypassing molecular replacement, and identifying potential protein interactors. By applying these AI tools in metagenomic studies, researchers can better understand the function of proteins more deeply. For example, they can uncover hidden microbial enzymes that may play roles in metabolism or disease.

Microbiome metabolomics is the study of the metabolites, or the small molecules produced by cellular metabolic processes (Bauermeister et al., 2022). These can be powerful markers for the downstream activity of microbes and provide a window into the chemistry and bioactivity of a community. They have profound implications in understanding the mechanistic link between microbes and their environment. However, metabolomics is a challenging and expensive modality that is rarely performed on stool samples, thus presenting numerous challenges for analysis. Despite these challenges, metabolomics techniques go well beyond identifying microbial species, and enable the inference of metabolic activities and functional roles.

Wang et al. (2023) introduced a method called mNODE to predict metabolomic profiles from microbial community data. This approach helped address the challenge of expensive and complex metabolomics experiments by using a ML technique called Neural Ordinary Differential Equations (NODE) (Chen et al., 2019), which is built on deep neural network models, to computationally predict the metabolomic profiles of microbiomes directly from metagenomic sequencing data. This method was tested on both synthetic and real-world data and shows better accuracy than existing methods. This approach can theoretically augment existing ubiquitous metagenomic sequencing data with AI predictions of its metabolites. As a result, it may reduce the need for expensive metabolomic experiments in many cases. It can also improve the value of older datasets, and accelerate microbiome research with fast, cost-effective metabolic predictions. This echoes the approach taken by PiCRUST (Langille et al., 2013) for functional predictions (using Bayesian ancestry reconstruction) using only limited marker gene (16S) compositional data. It is an example of predictive modality augmentation, which uses one type of (limited) data to generate first-order approximations of a different or more complex downstream data type.

Table 3 provides an overview of AI/ML applications across different stages of the metagenomic pipeline, including sequencing, quality control, assembly, and functional profiling. These advancements highlight the role of AI and ML.

**Table 3:** Applications of AI/ML across different stages of the metagenomic studies.

Stage	AI/ML application	Example Tools/Techniques
Sequencing	Improving base-calling accuracy for long-read technologies	DeepConsensus (Baid et al., 2023), MSRCall (Yeh & Lu, 2022)
Quality control	Automating error detection and improving QC processes	LongQC (Fukasawa et al., 2020), SHI7 (Al-Ghalith et al., 2018), SeqQscorer (Albrecht et al., 2021)
Assembly	Enhancing contig binning and MAG assembly	MetaBAT 2 (Kang et al., 2019), VAMB (Nissen et al., 2021)
Taxonomic profiling	Identifying microbial species and their abundance	TIPP2 (Shah et al., 2021), DNABERT-S (Zhou et al., 2025)
Functional profiling	Predicting microbial functions and metabolic pathways	mNODE (Wang et al., 2023), PICRUSt (Langille et al., 2013)

## Discussion

AI and ML have already transformed metagenomic research by addressing key challenges such as data complexity, QC, feature generation (through better assemblies and new foundation models), and downstream predictions of outcomes (or even entire data modalities). AI and ML can advance nearly every major stage of routine microbiome analysis, from data acquisition, to QC, to predictive modeling. To the latter point, AI and ML have demonstrated the potential to find patterns in complex microbial communities, enabling more accurate predictions about microbial functions, disease association, and environmental interactions.

As AI research advances and becomes more integrated into microbiome analysis, it is anticipated that more breakthroughs in metagenomics and microbiome research will be attributable to it. One such possibility for AI to make substantive improvements is the development of real-time microbiome analytics. With the advent of portable sequencing technologies like Oxford Nanopore’s MinION, combined with AI-driven base-calling and error correction tools, it is possible to perform real-time sequencing and analysis of microbial communities in the field. For example, real-time microbiome analysis could enable the identification of pathogens during a disease outbreak. It could also provide immediate feedback on the impact of dietary interventions on gut microbiota. These could both improve patients’ lives.

Another area that AI has the potential to improve is the integration of AI with multi-omics data. It is often desirable to combine metagenomics data with other omics types, such as transcriptomics, proteomics, metabolomics, and epigenomics. Doing so can allow for a more comprehensive systems-level understanding of microbial ecosystems (often in tandem with host biology and ‘omics). AI models are becoming able to natively integrate and analyze multi-omics data, and will thus be essential for more clearly studying the complex relationships between microbial communities and their hosts (Gupta et al., 2024).

However, it deserves to be mentioned that as AI and ML continue to be developed and become increasingly more capable, it is important to address their various challenges, particularly related to interpretability of the models and their inference process. It is often difficult to understand how a “black box” deep learning model arrives at its predictions. Additionally, as models become increasingly complex and are brought to feature spaces where the number of features far outstrips the sample size (the “p >> N” regime), overfitting becomes another concern. Thus, ensuring the interpretability and generalizability of any types of models will be critical for their widespread adoption.

While the main focus of this review is to discuss the latest advancements in AI across the metagenomic pipeline, it is important to also consider the ethical implications and data reproducibility of AI based biological research. Asnicar et al. (2024) highlight the principle of “garbage in, garbage out,” which means the results of a data processing pipeline, including AI/ML-based pipelines, are sensitive to input data quality. Models trained on datasets that have errors and noise are likely to produce misleading and unreliable results. In other words, the performance of machine learning models strongly depends on the quality of the data that are used for training. Additionally, both public metagenomic databases and AI bioinformatics tools are updated frequently. This can lead to different results when the same analyses are repeated at different times. For example, perfectly reproducing a study conducted a decade ago using R version 2.x can be difficult because it often requires downgrading software versions (Ziemann, Poulain & Bora, 2023), especially if packages used at the time have since been removed. To reduce data bias, studies should clearly report data source and preprocessing steps. Reproducibility can be also improved by using version-controlled workflows and documenting all software versions used throughout the analysis (from raw data QC to final figure generation). If randomization is used, a random seed should be set where possible to ensure reproducibility of results within a given software version. It is the scientific and ethical responsibility of researchers to ensure repeatable, reproducible results and transparency regarding the tools used.

## Conclusions

This review studies recent advances in artificial intelligence and machine learning across the metagenomic analysis pipeline. It highlights how these methods can handle complex data and reveal new biological insights. AI and ML are increasingly being integrated into the toolset used for analyzing microbiome data, and depending on the application currently represent both iterative refinements to existing tooling as well as sharper changes in capabilities. From slight increases to base quality or QC, to future real-time microbiome analyses and seamless integration of noisy multi-omics data, AI has the potential to drive both gradual evolution and breakthroughs in multiple fields, ultimately improving researchers’ understanding of human microbiota.

## Additional Information and Declarations

### Data Availability

The following information was supplied regarding data availability:

This is a literature review.
